# Periodontal diseases, potential mediators and development of liver fat content: a community-based large cohort

**DOI:** 10.3389/fmed.2025.1563459

**Published:** 2025-04-09

**Authors:** Bofu Liu, Yu Jia, Zhihan Gu, Yizhou Li, Yiheng Zhou, Yu Cao

**Affiliations:** ^1^Department of Emergency Medicine and Laboratory of Emergency Medicine, West China Hospital, Sichuan University, Chengdu, China; ^2^Disaster Medical Center, Sichuan University, Chengdu, China; ^3^General Practice Ward/International Medical Center Ward, General Practice Medical Center, West China Hospital, Sichuan University, Chengdu, China; ^4^State Key Laboratory of Oral Diseases, National Clinical Research Center for Oral Diseases, National Center of Stomatology, West China School of Stomatology, Sichuan University, Chengdu, China

**Keywords:** periodontal disease, periodontitis, liver fat content, liver proton density fat fraction, steatotic liver disease

## Abstract

**Background:**

A high level of liver fat content (LFC) is a key indicator of steatotic liver disease (SLD), reflecting its pathological essence. Periodontal disease (PD) recognized as a chronic inflammatory condition and cause a widespread adverse health impact. This study aims to investigate the relationship between PD and LFC development.

**Methods:**

In the UK Biobank, PD were gathered through a digital questionnaire, including gum pain, gum bleed, or teeth loose. LFC was measured by Fatty Liver Index (>60 indicates SLD) in cross-sectional analysis and by magnetic resonance imaging (quantified by Proton Density Fat Fraction, PDFF) in longitudinal analysis. Multivariable logistic and linear regression models were conducted to investigate the association of PD and LFC.

**Results:**

In cross-sectional analysis, 164,150 (37.4%) individuals were diagnosed with SLD, and PD showed a significant association with SLD (odds ratio: 1.104, 95% CI: 1.075–1.132). In prospective analysis, a total of 39,656 participants with a median follow-up of 10.3 years were included. PD showed an arithmetic mean difference of 0.091 in PDFF (95% CI: 0.047–0.139), with males exhibiting a stronger association than females (P for interaction <0.05). Significant mediating effects were observed for body mass index (19.58%), C-reactive protein (11.61%), blood glucose (6.70%), and healthy diet score (5.99%) between PD and PDFF (P for all <0.001).

**Conclusion:**

There was a pronounced correlation between PD and LFC, with males predominantly driving this link. This correlation may be partially mediated by body fat, inflammation, dietary habit, and insulin resistance.

## Introduction

Steatotic liver disease (SLD), characterized by liver fat content (LFC) of 5% or greater, is a hallmark of metabolic dysfunction-associated SLD (MASLD), previously known as non-alcoholic fatty liver disease (NAFLD) (1, 2). This condition has emerged as a prevalent and pressing global health issue. SLD is recognized for its potential to evolve into more severe liver pathologies, including metabolic dysfunction-associated steatohepatitis, cirrhosis, liver failure, and hepatocellular carcinoma, with projections indicating that MASLD may soon become the primary indication for liver transplantation (3, 4). Our prior research has delineated the trajectory of MASLD, highlighting LFC progression to a spectrum of extrahepatic systemic diseases and mortalities in a time-dependent sequence (5). Currently, the therapeutic arsenal lacks specific medications for LFC, remaining an essentially imperative to identify and understand the predictive factors and to develop targeted intervention strategies aimed at the progression of LFC.

Periodontal disease, a persistent bacterial infection, results in the degradation of the tissues that support teeth, including the alveolar bone (6). Recognized as a chronic inflammatory condition, periodontal disease has been linked to an elevated risk of various systemic illnesses, such as cardiovascular disease and cancer (7–9). The interplay between oral health and mental well-being, particularly SLD, is gaining increasing research interest. Despite numerous inquiries into the potential connections between periodontal disease and the risk of SLD, the consensus remains divided due to insufficient evidence to firmly establish these links (10–13). However, to our knowledge, there is no research exploring the relationship between periodontal disease and LFC, which has strong implications for providing direct evidence. Moreover, the existing research is often limited by its design, with many studies being cross-sectional, or with small sample size.

Furthermore, there is also a gap in underlying mechanisms that could explain how poor oral health might contribute to the LFC development. For example, evidence indicates that poor oral health is associated with elevated levels of inflammatory markers (14), such as C-reactive protein (CRP), which are known to be linked to various liver diseases (15). So, the potential factors were not well understood and require further elucidation. Therefore, this study aims to investigate the association between periodontal disease and LFC, and illustrate the potential mediators in these relationship.

## Materials and methods

### Study design and participants

The data in this study were collected from the UK Biobank. The U.K. Biobank included 502,357 participants aged 40 to 69 who were recruited through the United Kingdom National Health Service from April 2006 to December 2010. The UK Biobank study was approved by the North West Multi-Centre Research Ethics Committee (11/NW/0382). Informed consent was obtained from all participants. The Ethics Committee of West China Hospital, Sichuan University approved the use of the data.

**Figure 1:**
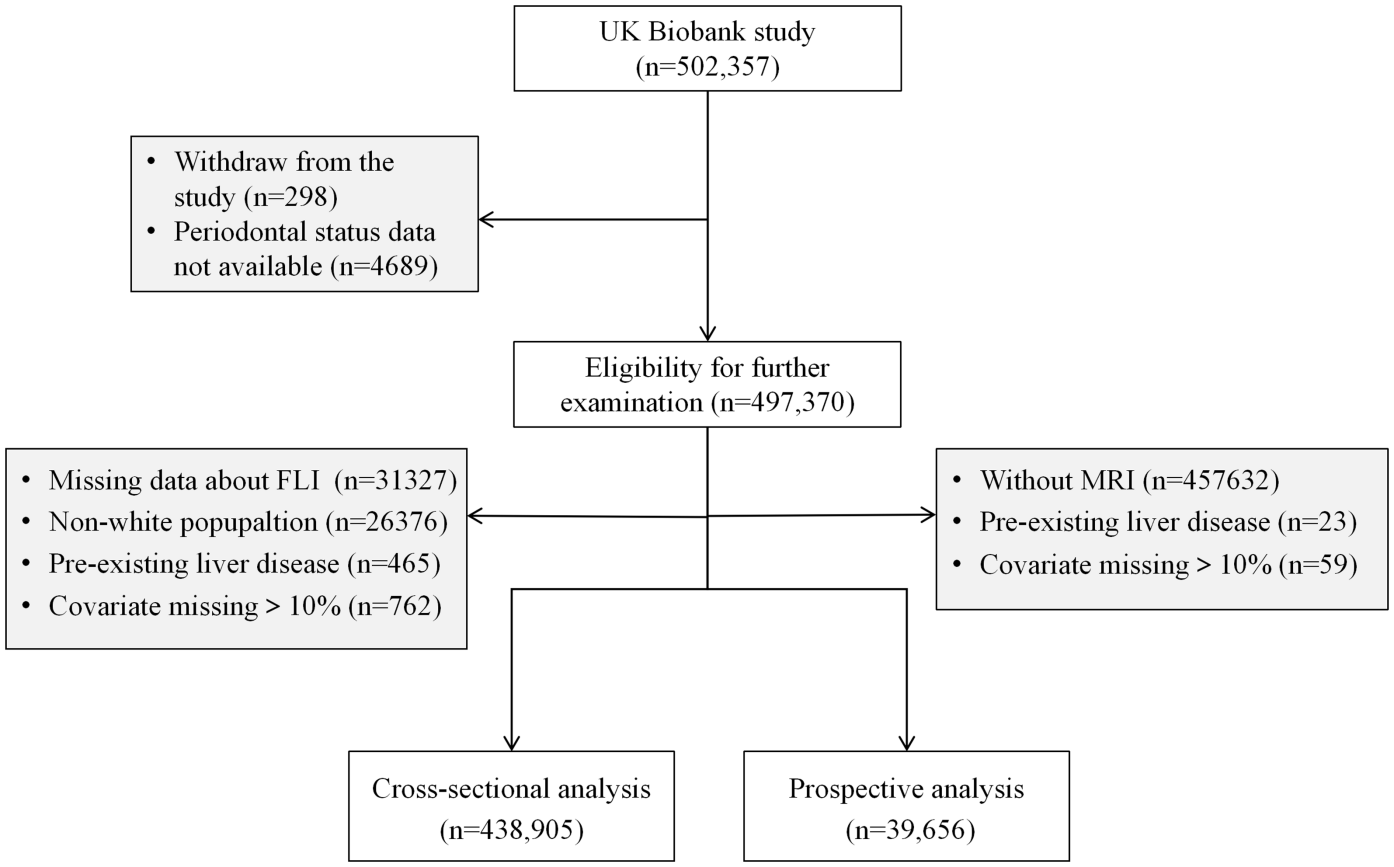
Participant flow chart.

This research encompasses both a cross-sectional and a prospective component. Eligibility for further examination was contingent upon participants providing valid periodontal status information and consenting to ongoing research engagement, totaling 497,370 individuals. For the cross-sectional segment, 438,905 participants were included after exclusions for the individuals without complete data necessary for the calculation of the Fatty Liver Index (FLI, *n* = 31,327), non-White participants (*n* = 26,376), those with pre-existing liver conditions (*n* = 465), and cases where >10% of covariates were missing (*n* = 762). In the prospective segment, the analysis included 39,656 participants after exclusions for the absence of MRI data (*n* = 457,632), presence of complicated pre-existing liver diseases (*n* = 23), and excessive missing covariate data (>10%, *n* = 59 instances). A visual representation of the participant selection process is depicted in Figure 1.

### Assessment of periodontal diseases

The self-assessment of periodontal conditions was conducted through a touchscreen-based questionnaire; a professional dental examination was not part of the process. Participants were queried about the presence of symptoms such as painful gums, bleeding gums, or loose teeth, with the option to select multiple symptoms or to indicate “none of the above.” There was also a provision for individuals to choose “prefer not to answer” if they wished. Individuals who reported experiencing one or more of these symptoms were categorized as being at an increased risk for periodontal disease. Conversely, those who reported an absence of these symptoms were considered to be at a low risk for developing periodontal issues. The self-assessment questionnaire effectively served as a predictive tool for periodontal disease, achieving area under the receiver operator characteristic curve of 0.82 according to previous study (16).

### Determination of liver fat content and SLD

Magnetic Resonance Imaging (MRI) serves as a sophisticated diagnostic tool for measuring Proton Density Fat Fraction (PDFF), which quantifies the proportion of protons associated with fat relative to the total protons within the liver. This non-invasive method allows for the precise determination of liver fat content, eliminating the need for a biopsy and offering accuracy rates approaching 100% (17).

In contrast, the Fatty Liver Index (FLI) represents a non-imaging, clinical scoring system utilized to evaluate the presence of steatosis. An FLI score of 60 or above is indicative of significant hepatic steatosis. The FLI takes into account various anthropometric and biochemical parameters, including Body Mass Index (BMI), triglyceride levels, gamma-glutamyl transferase (GGT) activity, and waist circumference, as per the criteria established in foundational research. With a cut-off value set at 60, the FLI has demonstrated a specificity of 86% and a sensitivity of 87% in identifying steatohepatitis (18). In this study, participants with FLI scores exceeding this threshold were classified as having steatohepatitis.

### Covariate assessment

Drawing on prior research and taking into account both exposure variables and study outcomes, we have pinpointed an array of covariates for consideration. These encompass demographic factors such as sex (male or female) and age, as well as socioeconomic status measured by the Townsend Deprivation Index. Lifestyle factors included patterns of alcohol consumption in grams per day, smoking habits categorized as never, former, or current smokers. Normal weight, overweight, and obesity were classified as body mass index (BMI) <25, 25–30, ≥30 kg/m^2^, respectively. Medical history, including the presence of hypertension and diabetes, is documented, along with biochemical measures like triglycerides, cholesterol, C-reactive protein levels, and sugar intake in grams per day. To assess dietary habits, a healthy diet score is derived from the consumption frequencies of beneficial food groups: fruits, vegetables, whole grains, fish, red meat, processed meat, and refined grains.

### Statistical analysis

In the crosssectional study, binary logistic regression models were used to investigate the association between periodontal disease and SLD, calculating odds ratios and 95% confidence intervals (CI). Four models were established: Model 1: unadjusted; Model 2: adjusted by sex and age; Model 3: adjusted by sex, age, deprivation index, alcohol intake, smoking status, hypertension, diabetes, triglyceride, and cholesterol; Model 4: adjusted by Model 3 plus BMI. In addition, we performed subgroup analyses stratified by age (<60 or ≥ 60 years), sex (male or female) and BMI (normal weight, overweight, or obesity). An interaction term was integrated into the model to appraise the uniformity of the observed associations.

In prospective investigation, we utilized multivariate linear regression models, as per established methodologies, to evaluate the correlation between periodontal status and PDFF. Given the skewed distribution of PDFF values, we employed the natural logarithm transformation to analyze the geometric means (19). The derived beta coefficients indicated the disparity in geometric means, and upon exponentiation, these coefficients translated into the ratio of arithmetic means. Additionally, the alteration in the geometric mean was articulated as a percentage change. Maintaining consistency with the cross-sectional study, the sequence of confounders considered in the prospective analysis paralleled that of the earlier work, and subgroup analyses were conducted.

Taking into account that periodontal disease significantly associated with several factors which serve as potential mediator for the LFC development, such as inflammation (C-reactive protein levels), dietary habits (healthy diet score and sugar intake), blood glucose, weight (BMI), blood lipid (cholesterol and triglyceride). Mediation and sensitivity analyses were conducted according to these factors. The outcomes of the path analysis were interpreted through standardized regression coefficients (*β*), which delineate both the direct and indirect impacts of periodontal disease on mortality rates (20). The mediation effect’s magnitude was ascertained by the ratio of the indirect path’s regression coefficient to the overall regression coefficient, providing a quantifiable measure of the influence conveyed through the mediating variable. We further refined our participant selection by excluding those reporting gum bleeding, a prevalent symptom within the general population—exceeding 50% according to a recent survey in the UK (21). This exclusion was based on the previous literature indicating the inaccuracy of using this symptom alone for diagnosing periodontal disease (22). Finally, we undertook a moderation analysis to discern the factors that influence the impact of periodontal disease on PDFF, ensuring a comprehensive examination of the interplay between these variables.

The statistical analysis was performed using SPSS (26.0 V, IBM Corp) and R (3.5.0 V) software. *p* < 0.05 was considered statistically significant.

## Results

### Cross-sectional analyses of relationship between periodontal status and SLD

#### Baseline characteristics

The baseline characteristics of the participants are shown in Supplementary Table S1. A total of 438,905 eligible participants with average age of 56.53 years were enrolled in this study. According to the FLI score, 164,150 (37.4%) individuals were diagnosed with SLD, in which group subjects had older age, higher BMI and cholesterol, higher proportion of males, hypertension, and diabetes, and lower high density lipoprotein levels compared to the other two groups.

#### Logistic regression analysis

Univariate logistic regression analysis showed that periodontal disease was significantly associated with SLD (Table 1), with OR values of 1.121 (95% CI: 1.104, 1.137). While, gum bleeding, tooth loss, and gum pain were also related to SLD (P for all <0.001). After fully adjusting for confounding factors, the associations of gum bleeding (OR: 1.059, 95% CI: 1.029, 1.090), gum pain (OR: 1.111, 95% CI: 1.061, 1.158), and periodontal disease (OR: 1.104, 95% CI: 1.075, 1.132) with SLD remain significant.

**Table 1:** Logistic regression models to analyse association between periodontal status and SLD.

Periodontal status	Model 1		Model 2		Model 3		Model 4	
	OR (95%Cl)	*p*	OR (95%Cl)	*p*	OR (95%Cl)	*p*	OR (95%Cl)	*p*
Gum bleed	1.039 (1.022, 1.057)	<0.001	1.222 (1.201, 1.244)	<0.001	1.054 (1.028, 1.080)	<0.001	1.059 (1.029, 1.090)	<0.001
Teeth loss	1.413 (1.376, 1.451)	<0.001	1.355 (1.318, 1.393)	<0.001	1.036 (0.996, 1.077)	0.080	1.018 (0.972, 1.066)	0.453
Gum pain	1.159 (1.103, 1.211)	<0.001	1.125 (1.089, 1.162)	<0.001	1.056 (1.002, 1.099)	0.014	1.111 (1.061, 1.158)	<0.001
Periodontal disease	1.121 (1.104, 1.137)	<0.001	1.272 (1.252, 1.292)	<0.001	1.059 (1.072, 1.120)	<0.001	1.104 (1.075, 1.132)	<0.001

Model 1: unadjusted; Model 2: adjusted by sex and age; Model 3: adjusted by sex, age, deprivation index, alcohol intake (g/day), smoking status (never, former, current), hypertension, diabetes, triglyceride, and cholesterol.; Model 4: adjusted by Model 3 plus BMI (kg/m^2^). SLD: steatotic liver disease.

In subgroup analysis according to BMI, age and sex, our results indicated that male has a stronger association between periodontal disease including gum bleeding, tooth loss, and periodontal disease with SLD (P for interaction <0.05, Supplementary Tables S2–S4). While, those associations were consistent in different BMI and age (P for interaction >0.05).

### Prospective analyses of relationship between periodontal status and LFC

#### Baseline characteristics

In the prospective study, a total of 39,656 participants with mean age of 55.01 years were involved during the median follow-up of 10.3 years. Among those participants, 7,053 (17.8%) individuals complicated with periodontal disease, and those individuals with lower age and male proportion, higher level of BMI and triglyceride, (*p* < 0.001), and higher proportion of current smokers, hypertension, and diabetes (Table 2).

**Table 2:** Baseline characteristics of participants with or without periodontal disease in prospective analysis.

Characteristics	Non-periodontal disease	Periodontal disease	*p*
Sample size, *n* (%)	32,603 (82.2%)	7,053 (17.8%)	
Male, *n* (%)	15,836 (49.2%)	2,869 (41.2%)	<0.001
Age (years)	55.23 ± 7.55	54.03 ± 7.38	<0.001
Deprivation index	−2.68 (−3.91,-0.60)	−2.33 (−3.73, 0.07)	<0.001
Alcohol drinker statues, *n* (%)			<0.001
Never	842 (2.6%)	172 (2.4%)	
Former	671 (2.1%)	154 (2.2%)	
Current	31,091 (95.3%)	6,726 (95.4%)	
Alcohol consume (g/day)	11.03 ± 10.12	11.44 ± 10.66	<0.001
Smoking status, *n* (%)			<0.001
Never	20,083 (61.6%)	4,052 (57.5%)	
Former	10,534 (32.3%)	2,541 (36.0%)	
Current	1987 (6.1%)	459 (6.5%)	
BMI (kg/m^2^)	26.40 ± 4.09	26.96 ± 4.42	<0.001
Hypertension, *n* (%)	6,251 (19.2%)	1,530 (21.7%)	<0.001
Diabetes, *n* (%)	1,242 (3.8%)	403 (5.7%)	<0.001
Cholesterol (mmol/L)	5.73 ± 1.09	5.74 ± 1.08	0.571
HDL-c (mmol/L)	1.48 ± 0.38	1.47 ± 0.38	0.234
LDL-c (mmol/L)	3.58 ± 0.83	3.58 ± 0.83	0.807
Triglyceride (mmol/L)	1.63 ± 0.95	1.65 ± 0.98	0.033

Data are expressed as mean ± standard deviation, *n* (%), or median (25th–75th). Deprivation index assigned by the postcode of participant location, which reflects the level of social deprivation in which the participant lives. BMI, body mass index; HDL-c, high-density lipoprotein cholesterol; LDL-c, low-density lipoprotein cholesterol.

#### Linear regression analysis

**Figure 2:**
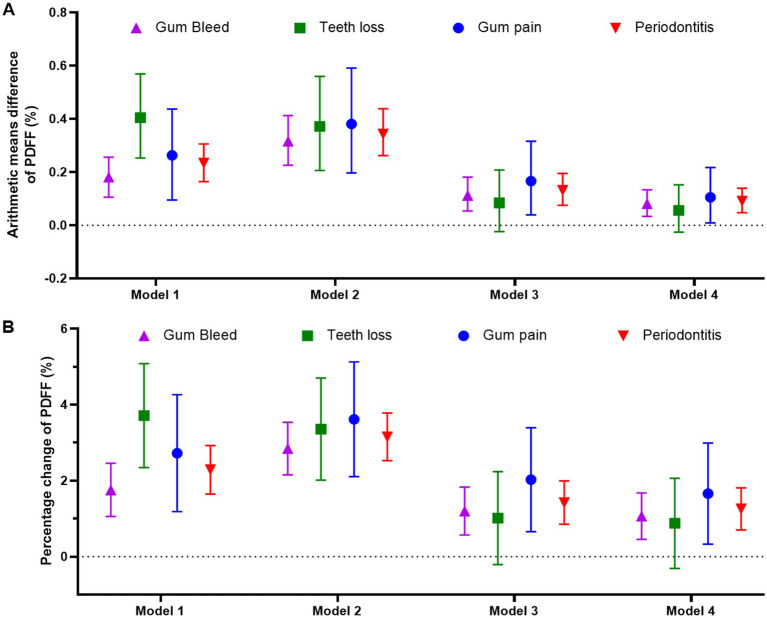
Linear regression models to analyze the association between periodontal status and arithmetic means difference of PDFF **(A)** or percentage change of PDFF **(B)**. Model 1: unadjusted; Model 2: adjusted by sex and age; Model 3: adjusted by sex, age, deprivation index, alcohol intake (g/day), smoking status (never, former, current), hypertension, diabetes, triglyceride, and cholesterol; Model 4: adjusted by Model 3 plus BMI (kg/m2). PDFF: proton density fat fraction.

In the fully adjusted linear regression models (Table S5), the arithmetic mean differences of PDFF for participants with periodontal disease compared to those without were as follows: 0.081 (95% CI: 0.033, 0.133) for gum bleeding, 0.105 (95% CI: 0.009, 0.217) for gum pain, and 0.091 (95% CI: 0.047, 0.139) for periodontal disease (Figure 2). These findings were mirrored in the percentage change of PDFF.

**Figure 3:**
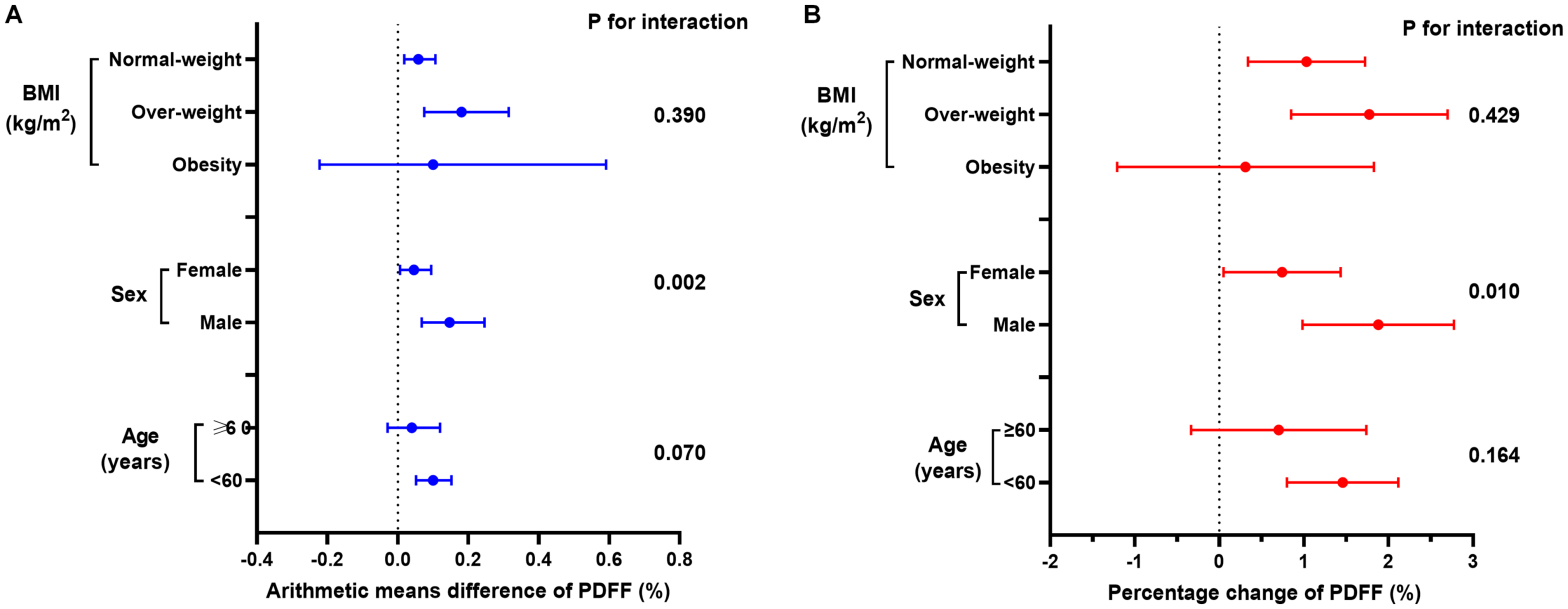
Association between periodontal disease and **(A)** arithmetic means difference of PDFF (%) or **(B)** percentage change of PDFF (%) in age, sex, and BMI subgroups. Linear regression models were adjusted by model 4.

Consistent with the cross-sectional analyses, males exhibited a more pronounced association for gum bleeding and periodontal disease, with a significant arithmetic mean difference in PDFF (P for interaction <0.05, Figure 3; Supplementary Tables S6–S8). In contrast, BMI and age did not significantly influence the relationship (P for interaction >0.05). The consistency of these results was observed for the percentage change in PDFF as well (Supplementary Table S9–S11).

#### Mediation, sensitivity, and moderation analyses

**Figure 4:**
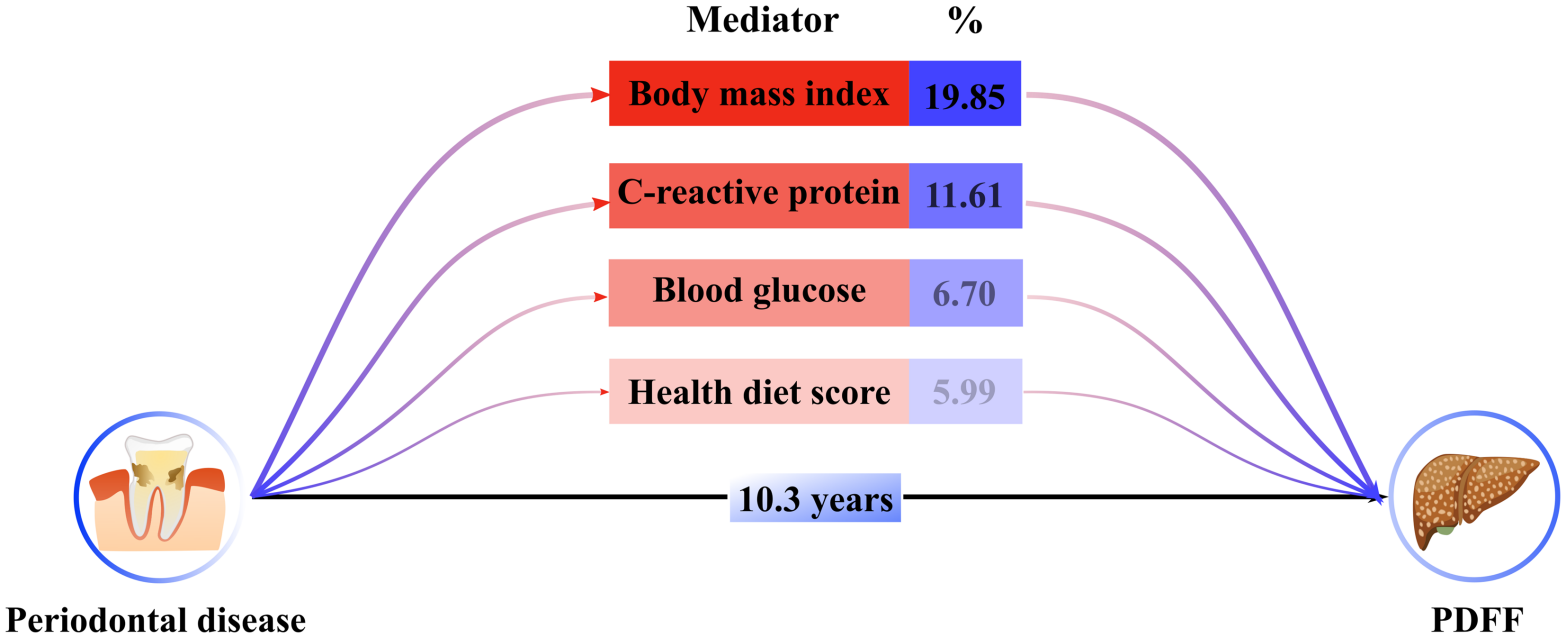
Estimated indirect effect percentage of different mediators on association between periodontitis and PDFF. A total of 1,000 iterations were performed for bootstrapping to estimate 95% bias-corrected confidence interval. Models were adjusted by model 4. The percentage was calculated by log (estimated indirect effect) /log (estimated total effect).

To elucidate the underlying mechanisms that could link periodontal disease to the progression of PDFF, we investigated the intermediary roles of various factors, including inflammation, blood glucose levels, body mass index, dietary habits, and lipid profiles. Utilizing structural equation modeling (Figure 4; Supplementary Table S12), we identified significant mediating effects of body mass index (19.58%), C-reactive protein levels (11.61%), blood glucose (6.70%), and healthy diet score (5.99%) between periodontal disease and PDFF (P for all <0.05).

In sensitivity analysis, the association between chronic pain and PDFF remained statistically significant after additional adjustment for the sugar intake, C-reactive protein, and health diet score, respectively (Supplementary Table S13). The conclusion remains consistent that the diagnosis of periodontal disease is limited to tooth loss and gum pain (Supplementary Table S14). Moderation analysis showed that female, BMI, and age weaken the associations, while alcohol consumption enhanced them (P for interaction <0.05, Supplementary Table S15). Significant interaction effect was not observed in the follow-up duration on this relationship (P for interaction: 0.743).

## Discussion

Our investigation, to the best of our current understanding, stands at the forefront of large-scale cohort studies, delving into the complex relationship between periodontal disease and LFC measured by MRI. Our findings indicate a robust positive correlation, suggesting that periodontal disease, especially gum bleed and gum pain, associated with the development of LFC. According to the results, in the cross-sectional study, periodontal disease was associated with a 10.4% increased risk of SLD. While, during the 10 years of follow-up, periodontal disease elevated absolute LFC of 0.09% and relative LFC of 1.26%. The consistency of these associations across various age groups and BMI categories, along with their resilience in sensitivity analyses, highlights the pivotal role of periodontal disease in the advancement of LFC. This underscores the imperative for serious consideration of interventions aimed at periodontal disease as a preventative measure against the escalation of LFC and the emergence of MASLD.

In this study, we observed a distinct result in terms of gender, by which the male have a significant stronger association between periodontal disease and LFC (arithmetic means difference of PDFF: 0.147 vs. 0.046). And this phenomenon had been reported by previous study (13). Feng et al. found that the prevalence of NAFLD was found to be significantly higher among males with severity of periodontal disease, an association not observed in females. This disparity may be explained by several potential mechanism. Initially, it’s noted that the incidence of SLD is considerably higher among males compared to females, with a marked difference observed in the percentages (50.7% vs. 24.1%) as we previous reported, potentially accounting for the noted discrepancy. Subsequently, variations in dietary habits between the sexes might also play a role in this divergence (23). Research indicates that women and men exhibit distinct dietary intake patterns and food preferences, which can influence the risk of developing NAFLD (24). Lastly, gender-based tendencies in health-damaging behaviors, such as smoking and alcohol consumption, are acknowledged as prevalent risk factors for NAFLD (25).

Dental plaque, which is partly made up of the cell walls of gram-negative bacteria, is frequently present in the human oral cavity, especially in individuals with periodontal disease (6). The escalation of endotoxins, such as lipopolysaccharides, and the rise of pro-inflammatory cytokines in the bloodstream can trigger a state of chronic, systemic, and low-grade inflammation (26). This type of inflammation is a key element in the development of obesity-related insulin resistance, which is a driving force in the onset and progression of NAFLD (27). The increase in serum levels of tumor necrosis factor-alpha (TNF-*α*), which can originate from LPS, can initiate and worsen insulin resistance. Hepatic insulin resistance is a recognized component in the pathogenesis of NAFLD (27, 28). Thus, it is plausible that the chronic low-grade inflammation and the intensification of insulin resistance establish a potential mechanistic connection between tooth loss and the development of NAFLD (27, 28). An additional mechanism linking oral health to overall health is dietary intake. Research has highlighted a correlation between oral health status and the quality of an individual’s diet (29–31). As the count of missing teeth rises, there is a tendency for diet quality to diminish. This is partly because the loss of teeth can affect the ability to chew and thus the consumption of certain foods, particularly those that require more effort to break down. Thus, plant-based foods, including fruits and vegetables, are highlighted as crucial mediators of periodontal disease that can influence disease progression (29, 30).

Building on our preliminary assumptions and existing scholarly work, we utilized a mediation effect model to investigate the underlying mechanisms by which periodontal disease might trigger the accumulation of LFC. Among the factors considered, C-reactive protein, blood glucose, body mass index, and health diet score stood out as substantial mediators influencing LFC development. This refined analysis pinpoints the role of inflammation, insulin resistance, body fat, and dietary habits in the pathway from periodontal disease to LFC accumulation. These findings underscore the multifactorial nature of LFC development and the significance of a holistic approach to managing periodontal health and its systemic impacts.

Our study is distinguished by several key strengths, such as its large-scale cohort, a designed cross-sectional and longitudinal methodology, and the use of MRI technology for the accurate assessment of LFC. We have considered a broad spectrum of potential confounding factors to fortify the analytical rigor of our research. Our comprehensive approach, encompassing factors like moderation analysis and intermediary variables, yields valuable insights for both intervention strategies and mechanistic dissection.

However, our study faces certain limitations. The UK Biobank cohort, with a selective response rate of 5.5%, may encounter the ‘healthy volunteer effect,’ potentially biasing the study’s applicability due to its correlation with the experience of periodontal disease. The reliance on self-reported measures of periodontal disease introduces the possibility of bias. The complexity of periodontal disease across biological, psychological, and social dimensions suggests that some confounding elements may not have been entirely accounted for. Lastly, the lack of a baseline PDFF in the dataset hinders the examination of the temporal evolution of PDFF.

## Conclusion

In summary, this study highlight a clear positive correlation between the periodontal disease and LFC, and this correlation is stronger in the male than the female. Moreover, we have identified a spectrum of factors—encompassing inflammation, body fat, diet habit, insulin resistance —that act as intermediaries in the relationship between periodontal disease and SLD. These factors also present themselves as potential targets for intervention to protect against SLD in periodontal disease sufferers. Further in-depth, high-caliber studies are needed to thoroughly clarify the fundamental mechanisms involved.

## Data Availability

The original contributions presented in the study are included in the article/Supplementary material, further inquiries can be directed to the corresponding author.
