# Supplementary material for: Periodontal diseases, potential mediators and development of liver fat content: a community-based large cohort
Source: Front Med (Lausanne). 2025 Apr 9;12:1563459. doi: 10.3389/fmed.2025.1563459 (PMC12014605; doi:10.3389/fmed.2025.1563459)
Supplement: Supplementary file 1 [file Table_1.docx]

Table S1. Baseline characteristics of participant by FLI in cross-sectional analysis.

| **Characteristics** |  | **FLI** |  |
| --- | --- | --- | --- |
|  | **FLI <30** | **30≤ FLI <60** | **FLI ≥60** |
| Sample size, n(%) | 155812 (35.5%) | 118943 (27.1%) | 164150 (37.4%) |
| Male, n(%) | 35642 (22.9%) | 59979 (50.5%) | 104508 (63.6%) |
| Age (years) | 55.14 ± 8.24 | 57.29 ± 8.02 | 57.30 ± 7.83 |
| Deprivation index | -2.30 (-3.73, 0.19) | -2.24 (-3.69, 0.36) | -1.87 (-3.50, 1.03) |
| Alcohol drinker statues, n (%) |  |  |  |
| Never | 6845 (4.4%) | 5387 (4.5%) | 7481 (4.6%) |
| Former | 5083 (3.3%) | 3942 (3.3%) | 6880 (4.2%) |
| Current | 143943 (92.3%) | 109412 (92.1%) | 149932 (91.3%) |
| Smoking status, n (%) |  |  |  |
| Never | 95365 (61.2%) | 65691 (55.3%) | 79087 (48.1%) |
| Former | 45540 (29.2%) | 40406 (34.0%) | 66156 (40.3%) |
| Current | 14965 (9.6%) | 12646 (10.6%) | 19049 (11.6%) |
| BMI (kg/m2 ) | 23.55 ± 2.37 | 26.84 ± 2.43 | 31.55 ± 4.52 |
| Hypertension, n (%) | 22755 (14.6%) | 30530 (25.7%) | 65885 (40.1%) |
| Diabetes, n (%) | 2574 (1.7%) | 4450 (3.7%) | 16269 (9.9%) |
| Cholesterol (mmol/L) | 5.63 ± 1.06 | 5.75 ± 1.14 | 5.71 ± 1.22 |
| HDL-c (mmol/L) | 1.67 ± 0.37 | 1.44 ± 0.33 | 1.26 ± 0.30 |
| LDL-c (mmol/L) | 3.42 ± 0.80 | 3.63 ± 0.87 | 3.63 ± 0.92 |
| Triglyceride (mmol/L) | 1.13 ± 0.49 | 1.63 ± 0.71 | 2.39 ± 1.19 |

Data are expressed as mean ± standard deviation, n (%), or median (25th-75th). Deprivation index assigned by the postcode of participant location, which reflects the level of social deprivation in which the participant lives. FLI: fatty liver index; BMI: body mass index; HDL-c: high-density lipoprotein cholesterol; LDL-c: low-density lipoprotein cholesterol.

Table S2. Subgroup analysis of association between periodontal status and FLI-SLD across different BMI.

| **Periodontal status** | **BMI** | **OR (95%Cl)** | **P value** | **P for interaction** |
| --- | --- | --- | --- | --- |
| **Gum bleed** | Total | 1.059 (1.029, 1.090) | <0.001 |  |
|  | Obesity | 1.004 (0.950, 1.060) | 0.887 |  |
|  | Over-weight | 1.094 (1.055, 1.133) | <0.001 | 0.356 |
|  | Normal-weight | 1.020 (0.908, 1.146) | 0.742 |  |
| **Teeth loss** | Total | 1.018 (0.972, 1.066) | 0.453 |  |
|  | Obesity | 1.038 (0.946,1.139) | 0.432 |  |
|  | Over-weight | 1.173 (1.002,1.374) | 0.048 | 0.439 |
|  | Normal-weight | 0.998 (0.943,1.056) | 0.953 |  |
| **Gum pain** | Total | 1.111 (1.061, 1.158) | <0.001 |  |
|  | Obesity | 1.041 (0.840, 1.208) | 0.665 |  |
|  | Over-weight | 1.121 (1.058, 1.180) | <0.001 | 0.605 |
|  | Normal-weight | 1.104 (1.009, 1.190 ) | 0.033 |  |
| **Periodontal disease** | Total | 1.104 (1.075, 1.132) | <0.001 |  |
|  | Obesity | 1.068 (1.015,1.123) | 0.011 |  |
|  | Over-weight | 1.121 (1.086,1.157) | <0.001 | 0.120 |
|  | Normal-weight | 1.133 (1.027,1.251) | 0.013 |  |

Logistic regression models were adjusted by sex, age, deprivation index, alcohol intake (g/day), smoking status (never, former, current), BMI (kg/m^2^), hypertension, diabetes, triglyceride, and cholesterol.

Table S3. Subgroup analysis of association between periodontal status and FLI-SLD across different age.

| **Periodontal status** |  | **Age<60 (n=282246)** | | **Age≥60 (n=215422)** | | **P for interaction** |
| --- | --- | --- | --- | --- | --- | --- |
|  |  | **OR (95%Cl)** | **P** | **OR (95%Cl)** | **P** |  |
| **Gum bleed** | Unadjusted | 1.115 (1.093, 1.138) | <0.001 | 1.000 (0.972, 1.029) | 0.991 | 0.157 |
|  | Adjusted | 1.039 (1.018, 1.061) | 0.007 | 1.005 (0.950, 1.062) | 0.871 |  |
| **Teeth loss** | Unadjusted | 1.525 (1.471, 1.581) | <0.001 | 1.277 (1.228, 1.329) | <0.001 | 0.091 |
|  | Adjusted | 1.063 (0.991, 1.130) | 0.085 | 0.970 (0.901, 1.046) | 0.431 |  |
| **Gum pain** | Unadjusted | 1.193 (1.135, 1.247) | <0.001 | 0.985 (0.938, 1.033) | 0.532 | 0.086 |
|  | Adjusted | 1.151 (1.078, 1.218) | <0.001 | 1.139 (1.055, 1.139) | 0.002 |  |
| **Periodontal disease** | Unadjusted | 1.183 (1.161, 1.205) | <0.001 | 1.090 (1.064, 1.116) | <0.001 | 0.071 |
|  | Adjusted | 1.097 (1.056, 1.140) | <0.001 | 1.060 (1.012, 1.110) | 0.014 |  |

Logistic regression models were adjusted by sex, age, deprivation index, alcohol intake (g/day), smoking status (never, former, current), BMI (kg/m^2^), hypertension, diabetes, triglyceride, and cholesterol.

Table S4. Subgroup analysis of association between periodontal status and FLI-SLD across different sex.

| **Periodontal status** |  | **Male (n=226926)** | | **Female (n=270742)** | | **P for interaction** |
| --- | --- | --- | --- | --- | --- | --- |
|  |  | **OR (95%Cl)** | **P** | **OR (95%Cl)** | **P** |  |
| **Gum bleed** | Unadjusted | 1.211 (1.180, 1.242) | <0.001 | 1.123 (1.097, 1.150) | <0.001 | <0.001 |
|  | Adjusted | 1.067 (1.017, 1.120) | 0.008 | 1.012 (0.967, 1.059) | 0.611 |  |
| **Teeth loss** | Unadjusted | 1.276 (1.228, 1.325) | <0.001 | 1.500 (1.441, 1.561) | <0.001 | <0.001 |
|  | Adjusted | 1.095 (1.026, 1.168) | <0.001 | 0.984 (0.954, 1.010) | 0.587 |  |
| **Gum pain** | Unadjusted | 1.176 (1.121, 1.230) | <0.001 | 1.211 (1.160, 1.263) | <0.001 | 0.263 |
|  | Adjusted | 1.157 (1.076, 1.231) | <0.001 | 1.122 (1.046, 1.192) | 0.002 |  |
| **Periodontal disease** | Unadjusted | 1.266 (1.237, 1.295) | <0.001 | 1.203 (1.178, 1.229) | <0.001 | 0.001 |
|  | Adjusted | 1.145 (1.098, 1.195) | <0.001 | 1.035 (0.993, 1.078) | 0.109 |  |

Logistic regression models were adjusted by sex, age, deprivation index, alcohol intake (g/day), smoking status (never, former, current), BMI (kg/m^2^), hypertension, diabetes, triglyceride, and cholesterol.

Table S5. Linear regression models to analyze the association between periodontal status and arithmetic means difference of PDFF or percentage change of PDFF.

| **Periodontal status** | **Model 1** | | **Model 2** | | **Model 3** | | **Model 4** | |
| --- | --- | --- | --- | --- | --- | --- | --- | --- |
|  | **Difference (95%Cl)** | **P** | **Difference (95%Cl)** | **P** | **Difference (95%Cl)** | **P** | **Difference (95%Cl)** | **P** |
| **Means difference of PDFF** | |  |  |  |  |  |  |  |
| Gum Bleed | 0.181 (0.105, 0.256) | <0.001 | 0.316 (0.226, 0.413) | <0.001 | 0.112 (0.054, 0.181) | <0.001 | 0.081 (0.033, 0.133) | <0.001 |
| Teeth loss | 0.405 (0.253, 0.569) | <0.001 | 0.372 (0.206, 0.56) | <0.001 | 0.084 (-0.024, 0.208) | 0.137 | 0.056 (-0.026, 0.152) | 0.192 |
| Gum pain | 0.263 (0.095, 0.437) | 0.002 | 0.381 (0.197, 0.591) | <0.001 | 0.166 (0.039, 0.316) | 0.009 | 0.105 (0.009, 0.217) | 0.031 |
| Periodontal disease | 0.234 (0.164, 0.306) | <0.001 | 0.343 (0.262, 0.438) | <0.001 | 0.131 (0.075, 0.195) | <0.001 | 0.091 (0.047, 0.139) | <0.001 |
| **Percentage change of PDFF** | |  |  |  |  |  |  |  |
| Gum Bleed | 1.757 (1.054, 2.461) | <0.001 | 2.846 (2.153, 3.539) | <0.001 | 1.199 (0.570, 1.828) | <0.001 | 1.067 (0.455, 1.679) | 0.001 |
| Teeth loss | 3.711 (2.343, 5.080) | <0.001 | 3.357 (2.013, 4.701) | <0.001 | 1.015 (-0.207, 2.236) | 0.103 | 0.876 (-0.313, 2.064) | 0.149 |
| Gum pain | 2.723 (1.183, 4.263) | 0.001 | 3.617 (2.106, 5.127) | <0.001 | 2.024 (0.657, 3.392) | 0.004 | 1.658 (0.327, 2.989) | 0.015 |
| Periodontal disease | 2.288 (1.649, 2.927) | <0.001 | 3.153 (2.525, 3.782) | <0.001 | 1.423 (0.852, 1.994) | <0.001 | 1.257 (0.701, 1.812) | <0.001 |

Model 1: unadjusted; Model 2: adjusted by sex and age; Model 3: adjusted by sex, age, deprivation index, alcohol intake (g/day), smoking status (never, former, current), hypertension, diabetes, triglyceride, and cholesterol; Model 4: adjusted by Model 3 plus BMI (kg/m^2^). PDFF: proton density fat fraction.

Table S6. Linear regression analyses of association between periodontal status and arithmetic means difference of PDFF in total, normal-weight, over-weight and obesity groups.

| **Periodontal status** | **BMI** | **Difference (95%Cl)** | **P value** | **P for interaction** |
| --- | --- | --- | --- | --- |
| **Gum bleed** | Total | 0.081 (0.033, 0.133) | <0.001 | 0.720 |
|  | Obesity | 0.054 (-0.295, 0.569) | 0.811 |  |
|  | Over-weight | 0.161 (0.048, 0.298) | 0.004 |  |
|  | Normal-weight | 0.054 (0.009, 0.107) | 0.017 |  |
| **Teeth loss** | Total | 0.056 (-0.026, 0.152) | 0.192 | 0.003 |
|  | Obesity | -0.613 (-1.077, 0.154) | 0.102 |  |
|  | Over-weight | 0.228 (0.021, 0.497) | 0.030 |  |
|  | Normal-weight | 0.064 (-0.026, 0.175) | 0.176 |  |
| **Gum pain** | Total | 0.105 (0.009, 0.217) | 0.031 | 0.823 |
|  | Obesity | 0.502 (-0.310, 1.772) | 0.271 |  |
|  | Over-weight | 0.131 (0.085, 0.190) | 0.028 |  |
|  | Normal-weight | 0.082 (0.010, 0.152) | 0.048 |  |
| **Periodontal disease** | Total | 0.091 (0.047, 0.139) | <0.001 | 0.390 |
|  | Obesity | 0.100 (-0.222, 0.591) | 0.592 |  |
|  | Over-weight | 0.181 (0.075, 0.315) | <0.001 |  |
|  | Normal-weight | 0.058 (0.018, 0.107) | 0.004 |  |

Linear regression models were adjusted by sex, age, deprivation index, alcohol intake (g/day), smoking status (never, former, current), BMI (kg/m^2^), hypertension, diabetes, triglyceride, and cholesterol.

Table S7. Linear regression analyses of association between periodontal status and arithmetic means difference of PDFF in younger and elderly.

| **Periodontal status** |  | **Age<60 (n=26930)** | | **Age≥60 (n=13083)** | | **P for interaction** |
| --- | --- | --- | --- | --- | --- | --- |
|  |  | **Difference (95%Cl)** | **P** | **Difference (95%Cl)** | **P** |  |
| **Gum bleed** | Unadjusted | 0.216 (0.126, 0.311) | <0.001 | 0.124 (-0.018, 0.276) | 0.086 | 0.076 |
|  | Adjusted | 0.089 (0.039, 0.147) | <0.001 | 0.015 (-0.063, 0.106) | 0.735 |  |
| **Teeth loss** | Unadjusted | 0.417 (0.208, 0.640) | <0.001 | 0.361 (0.142, 0.598) | 0.001 | 0.797 |
|  | Adjusted | 0.032 (-0.066, 0.148) | 0.553 | 0.085 (-0.032, 0.231) | 0.168 |  |
| **Gum pain** | unadjusted | 0.241 (0.043, 0.453) | <0.001 | 0.338 (0.037, 0.669) | 0.027 | 0.953 |
|  | adjusted | 0.121 (0.009, 0.216) | 0.024 | 0.103 (-0.061, 0.307) | 0.237 |  |
| **Periodontal disease** | unadjusted | 0.261 (0.176, 0.349) | <0.001 | 0.201 (0.077, 0.336) | 0.001 | 0.070 |
|  | adjusted | 0.100 (0.052, 0.152) | <0.001 | 0.040 (-0.029, 0.120) | 0.278 |  |

Linear regression models were adjusted by sex, age, deprivation index, alcohol intake (g/day), smoking status (never, former, current), BMI (kg/m^2^), hypertension, diabetes, triglyceride, and cholesterol.

Table S8. Linear regression analyses of association between periodontal status and arithmetic means difference of PDFF in males and females.

| **Periodontal status** |  | **Male (n=19119)** | | **Female (n=20894)** | | **P for interaction** |
| --- | --- | --- | --- | --- | --- | --- |
|  |  | **Difference (95%Cl)** | **P** | **Difference (95%Cl)** | **P** |  |
| **Gum bleed** | Unadjusted | 0.479 (0.332, 0.633) | <0.001 | 0.186 (0.104, 0.273) | <0.001 | 0.017 |
|  | Adjusted | 0.158 (0.068, 0.266) | <0.001 | 0.030 (-0.013, 0.081) | 0.173 |  |
| **Teeth loss** | Unadjusted | 0.289 (0.051, 0.546) | 0.016 | 0.443 (0.255, 0.643) | <0.001 | 0.500 |
|  | Adjusted | 0.050 (-0.081, 0.219) | 0.482 | 0.055 (-0.035, 0.164) | 0.252 |  |
| **Gum pain** | unadjusted | 0.395 (0.085, 0.728) | 0.011 | 0.330 (0.146, 0.531) | <0.001 | 0.652 |
|  | adjusted | 0.204 (0.026, 0.376) | 0.018 | 0.057 (-0.035, 0.169) | 0.238 |  |
| **Periodontal disease** | unadjusted | 0.449 (0.318, 0.582) | <0.001 | 0.248 (0.170, 0.330) | <0.001 | 0.002 |
|  | adjusted | 0.147 (0.068, 0.246) | <0.001 | 0.046 (0.006, 0.095) | 0.024 |  |

Linear regression models were adjusted by sex, age, deprivation index, alcohol intake (g/day), smoking status (never, former, current), BMI (kg/m^2^), hypertension, diabetes, triglyceride, and cholesterol.

Table S9. Linear regression analyses of association between periodontal status and percentage change of PDFF in total, obesity, over-weight and normal-weight groups.

| **Periodontal status** | **BMI** | **Difference (95%Cl)** | **P value** | **P for interaction** |
| --- | --- | --- | --- | --- |
| **Gum bleed** | Total | 1.067 (0.455, 1.679) | 0.001 | 0.652 |
|  | Obesity | 0.066 (-1.604, 1.735) | 0.939 |  |
|  | Over-weight | 1.497 (0.474, 2.519) | 0.004 |  |
|  | Normal-weight | 0.946 (0.183, 1.709) | 0.015 |  |
| **Teeth loss** | Total | 0.876 (-0.313, 2.064) | 0.149 | 0.006 |
|  | Obesity | -2.479 (-5.551, 0.592) | 0.114 |  |
|  | Over-weight | 2.220 (0.297, 4.142) | 0.024 |  |
|  | Normal-weight | 1.313 (-0.279, 2.905) | 0.106 |  |
| **Gum pain** | Total | 1.658 (0.327, 2.989) | 0.015 | 0.951 |
|  | Obesity | 2.357 (-1.171, 5.885) | 0.190 |  |
|  | Over-weight | 1.782 (0.741, 2.775) | 0.001 |  |
|  | Normal-weight | 1.661 (0.156, 3.152) | 0.036 |  |
| **Periodontal disease** | Total | 1.257 (0.701, 1.812) | <0.001 | 0.429 |
|  | Obesity | 0.309 (-1.210, 1.828) | 0.690 |  |
|  | Over-weight | 1.774 (0.848, 2.700) | <0.001 |  |
|  | Normal-weight | 1.031 (0.339, 1.723) | 0.004 |  |

Linear regression models were adjusted by sex, age, deprivation index, alcohol intake (g/day), smoking status (never, former, current), BMI (kg/m^2^), hypertension, diabetes, triglyceride, and cholesterol.

Table S10. Linear regression analyses association between periodontal status and percentage change of PDFF in younger and elderly.

| **Periodontal status** |  | **Age<60 (n=26930)** | | **Age≥60 (n=13083)** | | **P for interaction** |
| --- | --- | --- | --- | --- | --- | --- |
|  |  | **Difference (95%Cl)** | **P** | **Difference (95%Cl)** | **P** |  |
| **Gum bleed** | Unadjusted | 2.051 (1.223, 2.879) | <0.001 | 1.276 (-0.092, 2.645) | 0.068 | 0.126 |
|  | Adjusted | 1.293 (0.584, 2.002) | <0.001 | 0.267 (-0.951, 1.486) | 0.667 |  |
| **Teeth loss** | Unadjusted | 3.648 (1.789, 5.506) | <0.001 | 3.590 (1.602, 5.578) | <0.001 | 0.300 |
|  | Adjusted | 0.343 (-1.249, 1.935) | 0.673 | 1.521 (-0.248, 3.290) | 0.092 |  |
| **Gum pain** | unadjusted | 2.565 (0.713, 4.418) | 0.007 | 3.274 (0.492, 6.056) | 0.021 | 0.924 |
|  | adjusted | 1.671 (0.091, 3.252) | 0.038 | 1.574 (-0.895, 4.044) | 0.211 |  |
| **Periodontal disease** | unadjusted | 2.473 (1.705, 3.241) | <0.001 | 2.058 (0.895, 3.220) | 0.001 | 0.164 |
|  | adjusted | 1.458 (0.799, 2.116) | <0.001 | 0.702 (-0.334, 1.737) | 0.184 |  |

Linear regression models were adjusted by sex, age, deprivation index, alcohol intake (g/day), smoking status (never, former, current), BMI (kg/m^2^), hypertension, diabetes, triglyceride, and cholesterol.

Table S11. Linear regression analyses of association between periodontal statuss and percentage change of PDFF(%) in males and females.

| **Periodontal status** |  | **Male (n=19119)** | | **Female (n=20894)** | | **P for interaction** |
| --- | --- | --- | --- | --- | --- | --- |
|  |  | **Difference (95%Cl)** | **P** | **Difference (95%Cl)** | **P** |  |
| **Gum bleed** | Unadjusted | 3.903 (2.778, 5.027) | <0.001 | 1.948 (1.082, 2.814) | <0.001 | 0.004 |
|  | Adjusted | 1.918 (0.906, 2.931) | <0.001 | 0.450 (-0.301, 1.201) | 0.240 |  |
| **Teeth loss** | Unadjusted | 2.430 (0.462, 4.399) | 0.016 | 4.517 (2.688, 6.347) | <0.001 | 0.961 |
|  | Adjusted | 0.718 (-1.049, 2.486) | 0.426 | 1.057 (-0.535, 2.649) | 0.193 |  |
| **Gum pain** | unadjusted | 3.502 (1.009, 5.995) | 0.006 | 3.641 (1.769, 5.514) | <0.001 | 0.416 |
|  | adjusted | 2.292 (0.135, 4.319) | 0.006 | 1.187 (-0.433, 2.806) | 0.151 |  |
| **Periodontal disease** | unadjusted | 3.745 (2.748, 4.742) | <0.001 | 2.603 (1.804, 3.401) | <0.001 | 0.010 |
|  | adjusted | 1.879 (0.983, 2.775) | <0.001 | 0.741 (0.048, 1.435) | 0.036 |  |

Linear regression models were adjusted by sex, age, deprivation index, alcohol intake (g/day), smoking status (never, former, current), BMI (kg/m^2^), hypertension, diabetes, triglyceride, and cholesterol.

Table S12. Estimated indirect effect of different mediators on association between periodontal disease and PDFF.

| **Mediator** | **Arithmetic means difference of PDFF (95%Cl)** | | | **Mediated, (%)** |
| --- | --- | --- | --- | --- |
|  | **Total effect** | **Direct** | **Indirect** |  |
| C-reactive protein | 0.089 (0.045, 0.137) | 0.077 (0.035, 0.135) | 0.010 (0.005, 0.016) | 11.61* |
| Blood glucose | 0.094 (0.051, 0.145) | 0.085 (0.042, 0.129) | 0.006 (0.002, 0.011) | 6.70* |
| Body mass index | 0.091 (0.046, 0.140) | 0.072 (0.029, 0.097) | 0.018 (0.005, 0.016) | 19.58* |
| Health diet score | 0.096 (0.052, 0.146) | 0.084 (0.039, 0.147) | 0.006 (0.001, 0.015) | 5.99* |
| Cholesterol | 0.091 (0.047, 0.139) | 0.088 (0.042, 0.131) | 0.001 (-0.012, 0.015) | None  significant |
| Triglyceride | 0.091 (0.047, 0.139) | 0.086 (0.040, 0.140) | 0.005 (-0.021, 0.030) | None  significant |

A total of 1000 iterations were performed for bootstrapping to estimate 95% bias-corrected confidence interval. Models were adjusted by sex, age, deprivation index, alcohol intake (g/day), smoking status (never, former, current), BMI (kg/m2), hypertension, diabetes, triglyceride, and cholesterol. *P<0.001.

Overall effect indicates total (direct plus indirect) effect of periodontal disease on PDFF.

Indirect effect indicates the mediating effect of mediators (intermediate level) on association between periodontal disease and PDFF.

The percentage was calculated by log (estimated indirect effect)/ log (estimated total effect).

Table S13. Sensitivity analysis of the association between periodontal status and PDFF.

| **Periodontal status** | **Model 5** | | **Model 6** | | **Model 7** | | **Model 8** | |
| --- | --- | --- | --- | --- | --- | --- | --- | --- |
|  | **Difference (95%Cl)** | **P** | **Difference (95%Cl)** | **P** | **Difference (95%Cl)** | **P** | **Difference (95%Cl)** | **P** |
| **Means difference of PDFF** | |  |  |  |  |  |  |  |
| **Gum Bleed** | 0.086 (0.040, 0.122) | <0.001 | 0.092 (0.049, 0.153) | <0.001 | 0.078 (0.029, 0.151) | <0.001 | 0.088 (0.031, 0.148) | <0.001 |
| **Teeth loss** | 0.052 (-0.020, 0.134) | 0.183 | 0.059 (-0.041, 0.168) | 0.167 | 0.063 (-0.018, 0.174) | 0.198 | 0.050 (-0.012, 0.153) | 0.210 |
| **Gum pain** | 0.111 (0.094, 0.312) | 0.029 | 0.109 (0.098, 0.234) | 0.023 | 0.115 (0.065, 0.315) | 0.041 | 0.101 (0.004, 0.235) | 0.045 |
| **Periodontal disease** | 0.089 (0.042, 0.134) | <0.001 | 0.094 (0.045, 0.150) | <0.001 | 0.098 (0.050, 0.148) | <0.001 | 0.086 (0.039, 0.139) | <0.001 |
| **Percentage change of PDFF** | |  |  |  |  |  |  |  |
| **Gum Bleed** | 1.072 (0.512, 1.532) | <0.001 | 1.078 (0.613, 1.798) | <0.001 | 1.062 (0.552, 1.718) | 0.001 | 1.074 (0.589, 1.539) | <0.001 |
| **Teeth loss** | 0.870 (-0.293, 2.025) | 0.153 | 0.883 (-0.498, 2.384) | 0.162 | 0.890 (-0.335, 2.458) | 0.341 | 0.794 (-0.248, 1.998) | 0.523 |
| **Gum pain** | 1.672 (0.453, 2.863) | 0.010 | 1.668 (0.298, 2.296) | 0.012 | 1.723 (0.312, 2.823) | 0.011 | 1.598 (0.245, 3.369) | 0.029 |
| **Periodontal disease** | 1.234 (0.699, 1.770) | <0.001 | 1.342 (0.712, 1.974) | <0.001 | 1.352 (0.723, 1.991) | <0.001 | 1.229 (0.687, 1.774) | 0.001 |

Model 5: Model 4 plus sugar intake; Model 6: Model 4 plus C-reactive protein; Model 7: Model 4 plus health diet score; Model 8: Model 4 plus sugar intake, C-reactive protein, and health diet score.

Table S14. Sensitivity analysis of the association between periodontal status and PDFF.

| Adjustment | Means difference of PDFF | | Percentage change of PDFF | |
| --- | --- | --- | --- | --- |
|  | Difference (95%Cl) | P | Difference (95%Cl) | P |
| Model 4 | 0.073 (0.035, 0.106) | <0.001 | 1.167 (0.620, 1.700) | <0.001 |
| Model 5 | 0.075 (0.037, 0.107) | <0.001 | 1.258 (0.659, 1.873) | <0.001 |
| Model 6 | 0.062 (0.021, 0.105) | 0.001 | 0.996 (0.423, 1.420) | 0.002 |
| Model 7 | 0.070 (0.032, 0.118) | <0.001 | 1.228 (0.663, 1.878) | <0.001 |
| Model 8 | 0.060 (0.019, 0.105) | 0.003 | 0.991 (0.418, 1.991) | 0.002 |

Model 5: Model 4 plus sugar intake; Model 6: Model 4 plus C-reactive protein; Model 7: Model 4 plus health diet score; Model 8: Model 4 plus sugar intake, C-reactive protein, and health diet score.

Table S15. Moderating effect of confounding factors for association between periodontal disease and PDFF.

| **Adjusted factor** | **β** | **95% CI** | **P for interaction** |
| --- | --- | --- | --- |
| Female | -0.022 | -0.031, -0.013 | **0.001** |
| Age, (year) | -0.001 | -0.002, -0.000 | **0.031** |
| Deprivation index | 0.000 | -0.001, 0.002 | 0.156 |
| Alcohol consumption (g/d) | 0.001 | 0.000, 0.002 | **0.003** |
| Smoking status | -0.001 | -0.007, 0.004 | 0.642 |
| Body mass index (kg/m2 ) | -0.001 | -0.002, 0.000 | **<0.001** |
| Hypertension | -0.002 | -0.018, 0.014 | 0.781 |
| Diabetes | 0.006 | -0.009, 0.020 | 0.545 |
| Cholesterol (mmol/L) | 0.002 | -0.003, 0.007 | 0.697 |
| Triglyceride (mmol/L) | -0.003 | -0.008, 0.002 | 0.387 |
| Follow-up duration (year) | 0.000 | -0.002, 0.003 | 0.743 |

Linear regression models were adjusted by sex, age, deprivation index, alcohol intake (g/day), smoking status (never, former, current), BMI (kg/m^2^), hypertension, diabetes, triglyceride, and cholesterol.
